# Expression correlation attenuates within and between key signaling pathways in chronic kidney disease

**DOI:** 10.1186/s12920-020-00772-3

**Published:** 2020-09-21

**Authors:** Hui Yu, Danqian Chen, Olufunmilola Oyebamiji, Ying-Yong Zhao, Yan Guo

**Affiliations:** 1 Department of Internal Medicine, University of New Mexico, Albuquerque, NM 87131 USA; 2 Key Laboratory of Resource Biology and Biotechnology in Western China, School of Life Sciences, Northwest University, Xi’an, 710069 Shaanxi China

**Keywords:** Chronic kidney disease, Differential co-expression, Correlation attenuation, Pathway crosstalk

## Abstract

**Background:**

Compared to the conventional differential expression approach, differential coexpression analysis represents a different yet complementary perspective into diseased transcriptomes. In particular, global loss of transcriptome correlation was previously observed in aging mice, and a most recent study found genetic and environmental perturbations on human subjects tended to cause universal attenuation of transcriptome coherence. While methodological progresses surrounding differential coexpression have helped with research on several human diseases, there has not been an investigation of coexpression disruptions in chronic kidney disease (CKD) yet.

**Methods:**

RNA-seq was performed on total RNAs of kidney tissue samples from 140 CKD patients. A combination of differential coexpression methods were employed to analyze the transcriptome transition in CKD from the early, mild phase to the late, severe kidney damage phase.

**Results:**

We discovered a global expression correlation attenuation in CKD progression, with pathway *Regulation of nuclear SMAD2/3 signaling* demonstrating the most remarkable intra-pathway correlation rewiring. Moreover, the pathway *Signaling events mediated by focal adhesion kinase* displayed significantly weakened crosstalk with seven pathways, including *Regulation of nuclear SMAD2/3 signaling*. Well-known relevant genes, such as *ACTN4*, were characterized with widespread correlation disassociation with partners from a wide array of signaling pathways.

**Conclusions:**

Altogether, our analysis reported a global expression correlation attenuation within and between key signaling pathways in chronic kidney disease, and presented a list of vanishing hub genes and disrupted correlations within and between key signaling pathways, illuminating on the pathophysiological mechanisms of CKD progression.

## Background

Chronic kidney disease (CKD) entails gradual loss of kidney function leading to end-stage renal disease, precipitating the need for renal replacement therapies. The early stages of CKD, stages 1–2, have little signs or symptoms and the disease is often not detected until the later stages [1]. The risk of cardiovascular morbidity and mortality increases with the progression of CKD to stages 3–5. Omics-based approaches have emerged and explained the molecular differential expression during CKD progression. However, the disruption of gene coexpression in CKD remains obscure.

Whereas transcriptome data are most typically analyzed to find differentially expressed genes, an alternative analysis strategy [1–3] is gaining increasing popularity in helping decipher many human diseases. This emerging approach is focused on gene-gene connections/links and most concerned with the dynamical connections across comparative phenotypes. In 2005, a general framework for weighted gene co-expression network analysis was proposed [4], which was developed into a widely applied software WGCNA [5]. Many studies leveraged WGCNA to identify modules of coexpressed genes, which were constrained to have distinct expression levels between disease subjects and controls. Meanwhile, similar tools such as CoXpress, GSCA, and GSNCA [6] were invented with a direct goal of identifying extremely differentially coexpressed gene sets. Unlike the purely data-driven tool CoXpress [7], GSCA [8] and GSNCA incorporate gene function knowledge at the very beginning, and in consequence they only assess gene sets corresponding to functional units, such as Gene Ontology terms or cellular pathways in various senses. In addition to these set-wise analysis approaches, tools to mine individual genes and/or gene pairs with extreme differential coexpression are also available, including our own product DCGL [9]. Analytic overview of some of these aforementioned tools can be found in a recent review [10] on coexpression (and coexpression difference) methodologies.

The interactions among separate cellular pathways are referred to as pathway crosstalk [11–13], which may manifest notable changes from normal controls to disease subjects [14] and be informative for related drug development [15–17]. Most existing pipelines investigate the overlapping of (differentially expressed) genes between individual pathways, with or without consideration of the infrastructure of a protein-protein interaction network. We believe that the changed expression correlation relations (differential coexpression) born by individual gene pairs constitute a more context-specific network scaffold than a protein-protein interaction network, thus allowing for more relevant pathway crosstalk dynamics to be detected. Surprisingly, a pathway crosstalk analysis from the viewpoint of differential coexpression has not been undertaken.

While methodological progresses surrounding differential coexpression have helped with research on several human diseases [18–20], there has not been an investigation of coexpression disruptions in CKD patients yet. Therefore, we performed transcriptome profiling for 140 CKD patients dichotomized to early/late phases, and analyzed this CKD dataset as well as two related public datasets using a combination of pathway and pathway crosstalk analysis approaches centered upon differential coexpression. Strikingly, we discovered a pervasive disassociation of gene correlations in CKD progression, with pathway *Regulation of nuclear SMAD2/3 signaling* demonstrating the most remarkable intra-pathway correlation rewiring. In concordance with this global trend of correlation attenuation, 43 genes lost their hub statuses established in early CKD transcriptomes, including *ACTN4, ARF6, MAP2K7*, and *SRCAP*. Moreover, the pathway *Signaling events mediated by focal adhesion kinase* displayed significantly weakened crosstalk with seven pathways, including *Regulation of nuclear SMAD2/3 signaling*. Our analysis results proposed that vanishing hub genes and attenuated correlation within and between pathways may underpin the pathophysiological mechanisms of CKD advancement.

## Methods

### Human subjects

A total of 140 patients with different stages of CKD and a total of 25 control donors from Shaanxi Traditional Chinese Medicine Hospital, Xi’an No. 4 Hospital, and Baoji Central Hospital were included in our study. Patients with acute kidney injury, liver disease, active vasculitis, gastrointestinal pathology or cancer were excluded from the study. All study participants were ethnically Han Chinese. Patients (Demographic summary in Additional file 2: Table S1) were divided into CKD stage 1, 2, 3, 4 and 5 using creatinine-based estimated glomerular filtration rate (eGFR) equation [21]. We used the modified CKD-EPI equation to quantify eGFR [22]. No patients have received any treatment before diagnostic renal biopsy. Kidney tissue samples were collected from these patients through biopsy and stored in liquid nitrogen.

### RNA-Seq and data pre-processing

To reduce the cost of RNA-seq, samples were pooled into 33 pools, categorized roughly evenly to six stages (Normal, CKD 1–5). Total RNA extraction with ribosomal RNA removal (Ribo-Zero), RNA quality control, strand-specific library construction (Illumina), and 150 bp pair end RNA sequencing (Illumina) were conducted by Novogene. RNA-Seq data were quality controlled according to multi-perspective guideline [23] using QC3 [24]. Alignments were performed using STAR [25] against the GRCh38 reference genome, gene quantification was done using Cufflinks [26].

According to the National Kidney Foundation, eGFR is the best measurement for kidney function and is used to stage kidney disease. Patients with eGFR < 60 (CKD stage 3 to 5) are considered sufferring serious chronic kidney disease clinically. Also due to that the current differential coexpression approaches are generally incapable of handling minute sample size per category, we dichotomized all samples to an eGFR> 60 group (*n* = 16) and an eGFR < 60 group (*n* = 17), assigning normal, CKD1, and CKD2 to the former and CKD3–5 categories to the latter. Hence, by comparing between early and late CKD, we sought to elucidate cellular network rewiring mechanisms concurrent with the development of CKD from a mild, medically amenable stage to the severe end stage of kidney failure. This primary RNA-Seq dataset was denoted as CKD.

Two public microarray transcriptome datasets were also obtained for auxiliary analysis purpose. They were identified as GSE62792 and GSE37171 in Gene Expression Omnibus. GSE62792 [27] included 6 pooled samples for healthy volunteers and 12 pooled samples for CKD patients having uncertain etiology. The discovery phase of GSE37171 [28] consisted of 63 uremic patients and 20 healthy controls, manifesting a notably imbalanced class ratio. Because the significance of correlation coefficients is dependent on sample size, the paired correlation coefficients calculated from severely imbalanced normal group and disease group would be incomparable to each other. For this sake, we derived a balanced sub-dataset for GSE37171 where 21 randomly sampled patients were paired with the 20 controls. Of note, in the preliminary exploration of reproducibility of some differential coexpression methods, we generated three trial sub-datasets (GSE3.1, GSE3.2, and GSE3.3) from GSE37171, each encompassing one-third of the total patients (21 patients vs. 20 controls); we also generated five sub-datasets from GSE37171 (GSE3.1′, GSE3.2′, GSE3.3′, GSE3.4′, and GSE3.5′) which shared the 20 controls but each contained a different bootstrap sample of 21 patients.

To collapse multiple transcripts or probe sets to a single gene, we selected the entity that had the maximum median expression value across samples. All genes in a raw transcriptome dataset were sorted by their cross-sample median expression value, and those with relatively higher median expression values were kept in ensuing analyses. For the two microarray datasets, we used a threshold of 25% percentile; for the CKD dataset, we used the median (50% percentile) as the threshold. The three datasets contributed 28,574, 15,022, and 17,533 genes at this step, and an intersection among the three sources resulted in 11,400 shared genes. The number of considered genes eventually shrank to 2766 after constraining to well-defined pathways (see below). All correlation values were calculated using the Pearson method.

### Identification of differentially coexpressed genes and gene pairs

The method DCe from R package DCGL (v2.0) [9] was employed to identify differentially coexpressed links and differentially coexpressed genes from three kidney disease transcriptome datasets. For seeking of differentially coexpressed links, the coexpression network density (proportion of gene pairs deemed as coexpression links over all possible gene pairs) was set to 0.1, and 10% differentially coexpressed links was assumed a priori for running the Limit Fold Change model. For cutting off the differentially coexpressed gene list, we applied a threshold of q < 0.1.

### Identification of internally rewired pathways

From Pathway Commons [29], we obtained the union of pathways defined by PID [30], PANTHER [31], and INOH [32]. We included only these three database sources in order to minimize redundant pathways and deliberately bias towards signaling pathways. Identically named pathways from distinct sources were integrated into a singleton pathway by adopting the largest-sized gene set and adding additional gene members from a secondary source if that source contributed more than 70% shared gene members. After this pathway duplication ablation, we additionally examined gene overlapping between every pathway pair and ensured each pair of pathways have no more than 70% shared genes. This was done by iterating over pathways ordered by decreased set size, comparing the current pathway with each remaining pathway (of a smaller size) in terms of gene overlapping, and discarding the smaller-sized pathway if an over 70% overlapping was detected. With such integration and selection among raw pathways, we strived to achieve a minimum semantic redundancy among the compiled pathways. Finally, after constraining the pathways to the expression-measured genes and imposing a size limit of [5, 250], we came to a corpus of 369 pathways, which involved totally 2766 genes.

Gene Sets Net Correlations Analysis (GSNCA) [6] was exerted to assess the statistical significance of differential coexpression within each candidate pathways, where 1000 times’ permutation of sample class labels were implemented. Within one pathway, GSNCA summarizes the expression correlation profile for a gene with respect to all other peer genes, deriving a “weight” index for each gene. The weight vector, formed by weights of all member genes, is calculated for the two experimental conditions separately, and then the two weight vectors are incorporated into a distance statistic to indicate the degree of overall gene-gene rewiring within the candidate pathway. We utilized GSNCA to calculate the *p*-value for all 369 pathways in each of the three transcriptome datasets. We also let GSNCA output the hub gene and the schematic gene wiring network for each pathway. In GSNCA’s terminology, a hub is a gene that has the maximum weight, and the gene wiring network is the union of the first and second minimum spanning trees, which were identified through minimizing the total path length (sum of correlation distances). A hub gene and a pathway intra-wiring network are associated with one pathway under one experimental condition.

### Discovery of disrupted pathway crosstalk

Huang et al. devised an algorithm [33] to identify characteristic sub-pathway network (CSPN) through appreciating significantly abundant inter-pathway gene-gene links. In two schizophrenia studies [34, 35], CSPN was leveraged to delineate pathway crosstalk maps in principle of over-represented protein-protein interactions. Here, rather than using the conventional protein-protein interaction network, we let the differentially coexpressed links form the scaffold network. The differentially coexpressed links out of the RNA-seq dataset served as the primary network source, whereas a union network of differentially coexpressed links from the three transcriptome datasets was also analyzed for verification purpose. CSPN evaluated all pairwise connections among the significantly rewired pathways flowed from the upstream GSNCA analysis. Finally, inter-pathway links with *p* < 0.05 were kept in the pathway crosstalk map.

## Results

### Reproducibility of the differential coexpression approach

**Fig. 1:**
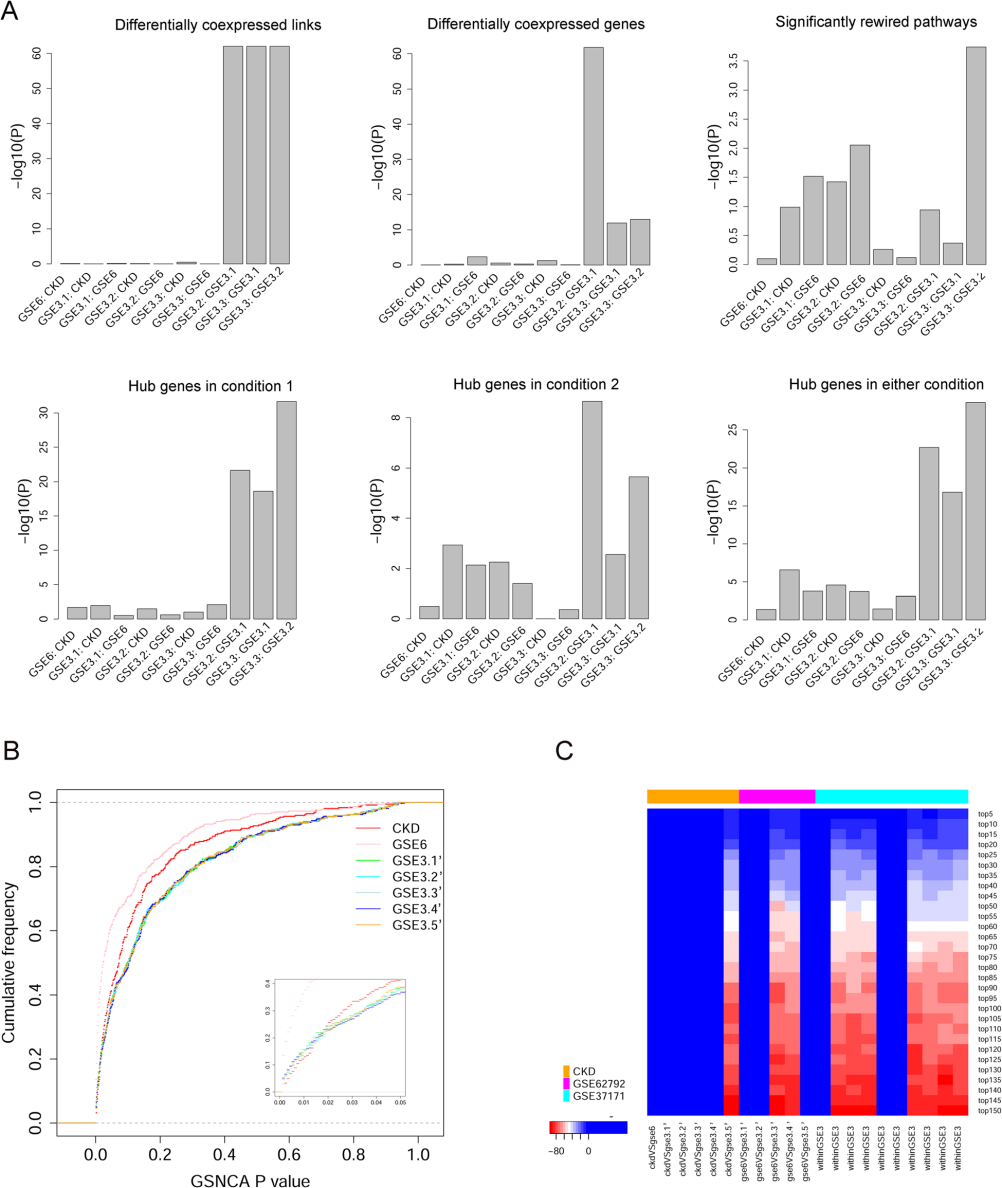
Overlap of resultant entity across kidney transcriptome datasets of different sources. **a** statistical significance of set intersection between dataset pairs. Hypergeometric probability model was employed to calculate the *p*-value of obtaining the actual or a greater number of shared entities. Bar height symbolizes the inverse of *p*-values, thus the higher the more significant. GSE6, GSE62792; GSE3, GSE37171; GSE3.x, a derived dataset originating from GSE37171, with balanced sample sizes (20 vs. 21). **b** empirical cumulative density function curves for the 369 pathway-wise p-values determined by GSNCA in each dataset. The 21 disease samples in each GSE3.x dataset were randomly selected from the whole set of 63 samples, and these selected disease samples may share in part among the five derived datasets. **c** statistical significance of intersection between top-ranking pathways from different datasets. Top-ranking pathways were gradually enlarged from 5 to 150 (40.1% of all pathways) at an interval of 5 (row labels). Color shade is proportional to log10(p), where p is the p-value calculated under hypergeometric probability model. Red signifies high portion of intersection entities unexpected by random cases

From each of five datasets (CKD, GSE62792, GSE3.1, GSE3.2, and GSE3.3; see Methods for dataset explanation), we identified differentially coexpressed links, differentially coexpressed genes, significantly rewired pathways, and pathway hub genes. We assessed the overlapping significance among five data sources using the hypergeometric test, where the total number of candidate entities were 369 for pathways, 2766 for genes, and 3,823,995 $\left({C}_{2766}^2\right)$ for gene links. The extremity of *p* values out of the hypergeometric tests was visualized in barplots (Fig. 1a). In all facets except for rewired pathways, the three repetitive sub-datasets derived from GSE37171 showed significantly similar results; by contrast, agreement among the three distinct sources (CKD, GSE62792, and GSE37171) was generally insignificant. We were concerned by the fact that the significant pathway lists mined from three repetitive sub-datasets did not display significant agreement with each other, so we generated another five sub-datasets from GSE37171 by matching the 20 controls with a bootstrap subset of 21 patients each time. The five bootstrap-sampled sub-datasets had less conspicuous pathway *p*-values compared with datasets CKD and GSE62792 (Fig. 1b). When we delimited a fixed number of top-ranking pathways, significant agreement among datasets, especially among repetitive sub-datasets, arose (Fig. 1c).

Given these reproducibility results from repetitive sub-datasets, we believed those differential coexpression methods were capable of yielding statistically stable results when different yet same-natured samples were recruited to represent a phenotype. The much lower (and mostly insignificant) result consistency among three distinct data sources prompted us to speculate that our three datasets bore considerable disparity in their molecular mechanisms, despite all being related to kidney diseases. In our formal workflow, we primarily focused on the RNA-seq dataset (denoted “CKD”), integrating GSE62792 and GSE37171 as auxiliary data only in partial analyses. In particular, with respect to the less mutually consistent pathway-level results, we resorted to all three datasets to compile a list of focused pathways that were significantly rewired per two data sources or more.

### Pervasive disassociation of gene correlation

**Fig. 2:**
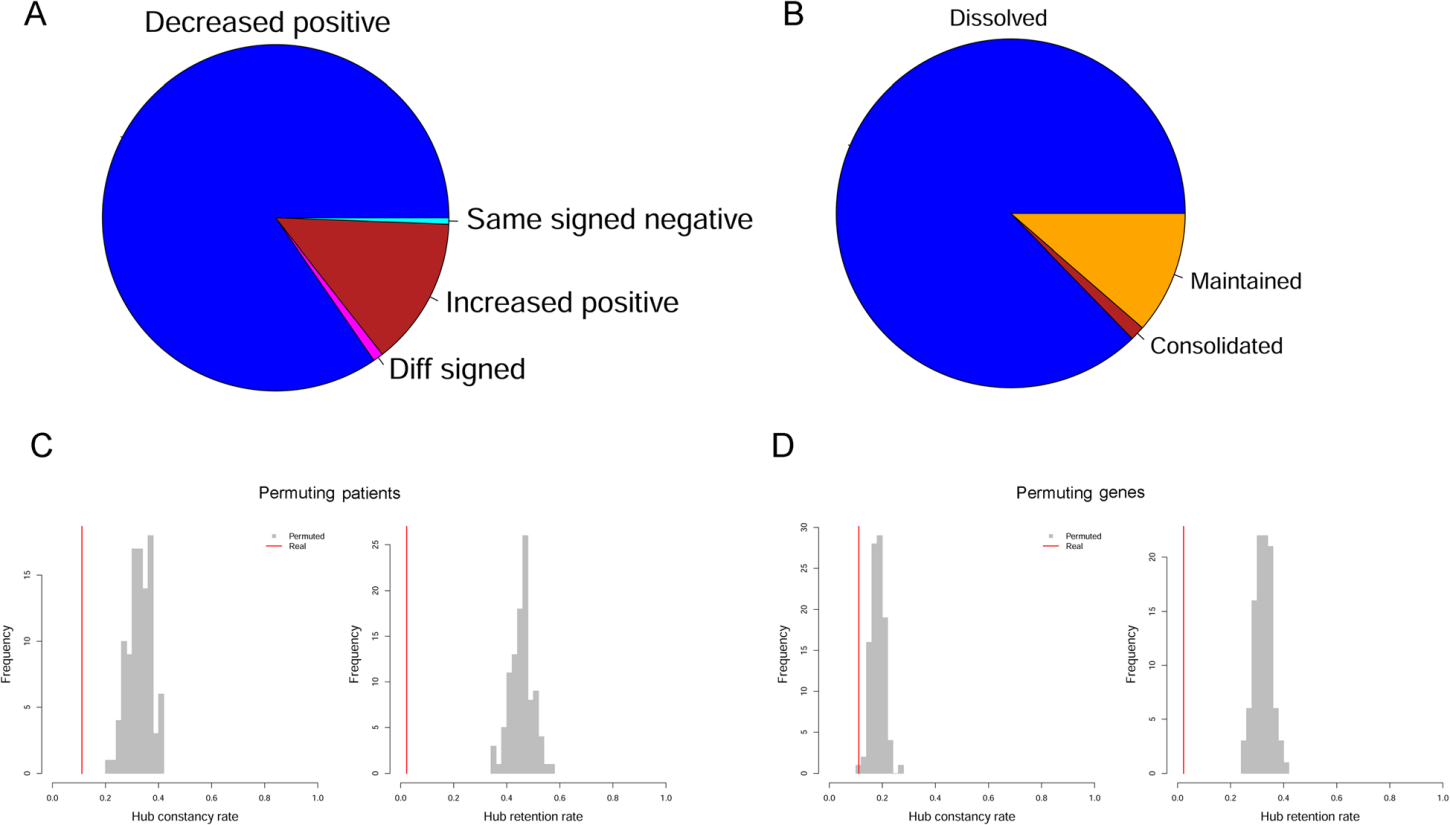
Global expression correlation attenuation and extremely low hub retention of pathways. **a** breakdown of differentially co-expressed gene links (DCLs). Each DCL is characterized with a pair of correlation values corresponding to the two comparator conditions, respectively, and DCLs are categorized into four types on account of the signs and changing trend of the paired correlation values. Diff signed, DCLs of two extreme correlation values in opposite signs. Same signed negative, DCLs of two negative correlation values. Increased positive, DCLs showing correlation increment toward extreme positive values. Decreased positive, DCLs showing correlation decrease from extreme positive values. **b** breakdown of pathways by predominant correlation change direction. Dissolved, more gene pairs have decreased correlation. Consolidated, more gene pairs have increased correlation. Maintained, even share of gene pairs with increased correlation and gene pairs with decreased correlation. **c** one hundred times of random permutation of patients’ class labels were performed and GSNCA was implemented on the permutated datasets, with respect to all 369 covered pathways. The real hub constancy rate (3/27) and hub retention rate (1/44) was compared against the empirical distributions resulting from permutations. **d** hub constancy rates and hub retention rates in real data analysis (red line) and permuted analyses (grey histogram), where one hundred times of random permutation of patients’ pathway annotations preceded GSNCA running. Technically, permuting patients’ pathway annotations was equivalent to shuffling the gene-to-pathway mapping relations, thus achieving random organization of genes to meaningless pseudo-pathways while maintaining the same pathway size profile. The real hub constancy rate (3/27) and hub retention rate (1/44) was compared against the empirical distributions resulting from permutations

DCGL categorized its identified differentially coexpressed links according to the signs of correlation values in the two compared conditions. From dataset CKD, we noted an overwhelming dominance (85.4%) of decreased-positive links (Fig. 2a), though this pattern was not apparent in results out of GSE62792 and GSE37171. Relatedly, we intuitively assorted the candidate pathways into three categories based on the predominant correlation change direction: consolidated (incremental correlations outweighed decremental correlations), dissolved (decremental correlations outweighed incremental correlations), and maintained (balanced constitution of incremental/decremental correlations). In accordance with the disproportional constitution of differentially coexpressed links, a majority (87.3%) of the candidate pathways were found dissolved from early CKD to late CKD (Fig. 2b).

**Fig. 3:**
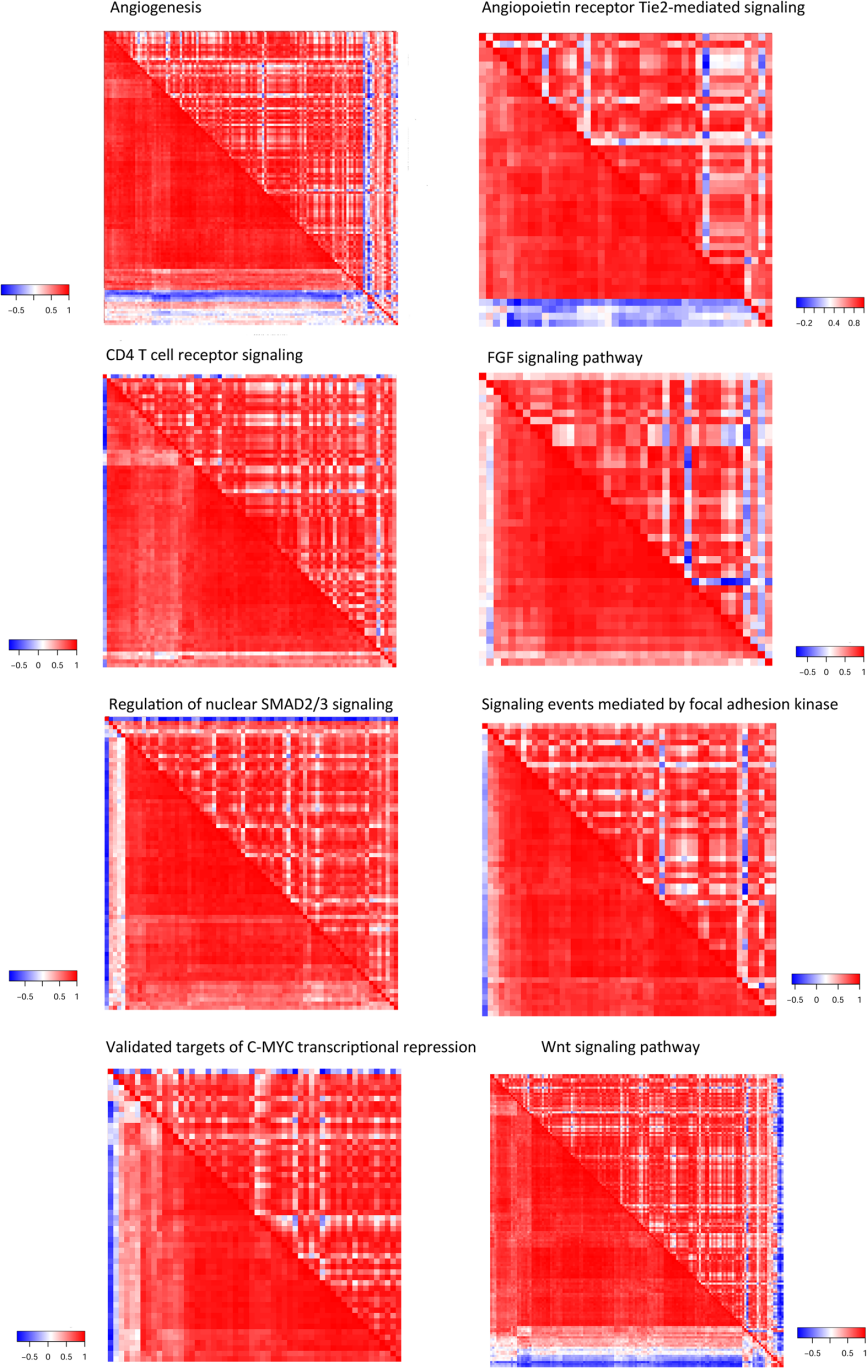
Universal correlation attenuation within individual pathways. Rows and columns represent genes of the concerned pathway, arranged in identical order. Cells denote the expression correlation values between the row gene and the column gene, with the lower triangle and the upper triangle indicating the early CKD and late CKD phenotypes, respectively

From the three transcriptome datasets, 46, 141, and 20 pathways stood out as significantly rewired pathways from CKD, GSE62792, and GSE37171, respectively. Each represented a considerable portion of the total candidate pathways, yet not showing significant overlapping with each other. We adopted Fisher’s combined probability test to aggregate the *p* values from individual datasets, and compiled 27 focused pathways (Table 1) which were found significantly rewired per at least two datasets (*p* < 0.01) and had an aggregate p below 0.01. In decreasing aggregate p, *Regulation of nuclear SMAD2/3 signaling* (originating from PID) emerges as the most noteworthy pathway, showing p values 0.012, 0.0010, and 0.0020 in CKD, GSE62792, and GSE37171, respectively. In concordance with the pervasive disassociation trend between genes, most of these focused pathways displayed far-flung correlation loss among their member genes (Fig. 3 and Additional file 1: Fig. S1).

**Table 1:** Twenty-seven focused pathways that significantly changed the internal gene-gene expression correlation in CKD advancement

Pathway	Aggregated *p*	CKD RNA-Seq			GSE62792			GSE37171		
		*p*	Early	Late	*p*	Normal	Disease	*p*	Normal	Disease
Regulation of nuclear SMAD2/3 signaling	4.13E-06	0.012	*FOXO1*	*MAX*	0.001	*HNF4A*	*TCF3*	0.002	*SMAD2*	*FOXO1*
CD4 T cell receptor signaling	4.74E-06	0.014	*HLA-DPB1*	*NFKB1*	0.001	*HLA-DPA1*	*PPP3CC*	0.002	*MAP 3 K7*	*CD247*
TCR signaling in naive CD4+ T cells	5.93E-06	0.018	*PTPRC*	*CBL*	0.001	*CD3D*	*IKBKB*	0.002	*RASSF5*	*CD247*
IL2 signaling events mediated by STAT5	1.07E-05	0.001	*BCL2*	*BCL2*	0.010	*GRB2*	*CDK6*	0.007	*SHC1*	*CCND2*
Angiopoietin receptor Tie2-mediated signaling	1.82E-05	0.001	*FOXO1*	*NFKB1*	0.002	*AKT1*	*RAC1*	0.064	*FOXO1*	*MAPK1*
TRAIL signaling pathway	1.87E-05	0.033	*TNFSF10*	*TNFRSF10D*	0.002	*FADD*	*DAP3*	0.002	*PIK3CA*	*MAPK1*
Signaling events mediated by HDAC Class II	2.46E-05	0.030	*HSP90AA1*	*GNB1*	0.001	*NUP214*	*BCOR*	0.006	*GNB1*	*RAN*
Neurotrophic factor-mediated Trk receptor signaling	3.22E-05	0.061	*STAT3*	*RAP1B*	0.001	*MAPK3*	*RAPGEF1*	0.004	*RHOA*	*RHOA*
Canonical Wnt signaling pathway Diagram	3.41E-05	0.009	*GSK3B*	*FZD5*	0.001	*FRAT1*	*DVL3*	0.029	*WNT11*	*EP300*
Cellular roles of Anthrax toxin	5.90E-05	0.008	*MAP 2 K7*	*MAP 2 K1*	0.001	*MAPK3*	*TNF*	0.061	*MAP 2 K4*	*MAPK1*
Methionine and Cysteine metabolism	6.62E-05	0.556	*MTR*	*MTR*	0.001	*LDHA*	*AHCY*	0.001	*GOT2*	*LDHB*
mRNA splicing	6.65E-05	0.040	*PRPF3*	*SNRPA*	0.007	*PRPF3*	*PRPF3*	0.002	*PRPF3*	*SNRPA*
Integrin signaling pathway	6.94E-05	0.006	*ARF6*	*ILK*	0.001	*ITGAX*	*ITGB7*	0.098	*RHOA*	*RHOA*
Validated nuclear estrogen receptor alpha network	7.38E-05	0.158	*SET*	*XBP1*	0.004	*LCOR*	*HDAC1*	0.001	*DDX17*	*XBP1*
Wnt signaling pathway	7.87E-05	0.010	*SRCAP*	*HDAC3*	0.001	*PRKCD*	*CSNK2A2*	0.068	*GNB1*	*PPP2R5C*
Arf6 signaling events	0.00010	0.116	*EGFR*	*GNA14*	0.002	*ACAP2*	*CYTH3*	0.004	*GNAQ*	*NCK1*
Alpha4 beta1 integrin signaling events	0.00012	0.095	*ARF6*	*CD14*	0.006	*CDC42*	*CD14*	0.002	*YWHAZ*	*ITGA4*
FGF signaling pathway	0.00012	0.009	*ETS2*	*ETS2*	0.004	*SPI1*	*PLCG1*	0.032	*MAPK1*	*MAPK1*
Syndecan-4-mediated signaling events	0.00012	0.003	*THBS1*	*CCL5*	0.055	*CXCR4*	*GIPC1*	0.007	*ACTN1*	*RHOA*
AP-1 transcription factor network	0.00017	0.003	*HLA-A*	*ETS1*	0.009	*MMP9*	*TRIP6*	0.063	*CREB1*	*CDKN1B*
Signaling events mediated by focal adhesion kinase	0.00021	0.009	*ACTN4*	*RAP1B*	0.001	*ROCK2*	*ARHGEF7*	0.233	*RHOA*	*RHOA*
a6b1 and a6b4 Integrin signaling	0.00030	0.002	*YWHAZ*	*YWHAG*	0.005	*AKT1*	*PIK3R1*	0.319	*PIK3R1*	*YWHAQ*
ATM pathway	0.00059	0.003	*ATM*	*ABL1*	0.005	*TOP3A*	*ATM*	0.472	*TRIM28*	*SMC1A*
Validated targets of C-MYC transcriptional repression	0.00062	0.009	*CDKN1B*	*CEBPD*	0.001	*CREB1*	*HDAC1*	0.833	*ALDH9A1*	*EP300*
Apoptosis signaling pathway	0.00063	0.005	*TNFRSF1A*	*ATF4*	0.002	*BIRC3*	*LTB*	0.767	*PIK3CA*	*HSPA5*
Angiogenesis	0.00065	0.010	*MAPKAPK2*	*CRKL*	0.005	*PRKCD*	*GRAP*	0.161	*GSK3B*	*RHOA*
Cadherin signaling pathway	0.00072	0.009	*CSNK2A2*	*FZD5*	0.006	*ACTA2*	*LEF1*	0.167	*WNT11*	*EGFR*

### Vanishing hubs in global correlation attenuation

For a phenotype, early CKD or late CKD, GSNCA identified one hub gene for each pathway, which can be intuitively conjectured as the center of the gene correlation wiring network (a more technical explanation was given in Methods). The significantly rewired pathways featured 40 and 40 hub genes in early CKD and late CKD, respectively, which shared only six genes. Of the 27 focused pathways out of combined evidence from three datasets, only three had a constant hub in early CKD and late CKD (Table 1). Since a global correlation loss was found to pervade the CKD advancement transcriptomes, we believed the vanishing hubs be more relevant to CKD advancement than emerging hubs. Indeed, with respect to all 369 candidate pathways, more early-phase hubs were identified as differentially coexpressed genes than late-phase hubs (44 vs. 7, precisely). Of the 44 differentially coexpressed, early-phase hub genes, only one gene (*GCLM*) maintained its hub status in CKD advancement; all other 43 genes (Additional file 2: Table S2) became “vanishing hubs” as CKD advanced to end stage. Fourteen vanishing hub genes were found differentially expressed among the six disease groups when False Discovery Rate (FDR) was controlled below 0.3 (Additional file 1: Fig. S2).

We evaluated the statistical significance of the observed hub constancy rate (3/27) and hub retention rate (1/44) by comparing them against two empirical distributions, which resulted from random permutation of patients’ class labels (Fig. 2c) or genes’ pathway annotations (Fig. 2d). The permutation experiments indicated the observed hub constancy rate (3/27) and hub retention rate (1/44) were significantly rare in random cases (*p* ≤ 0.01).

**Fig. 4:**
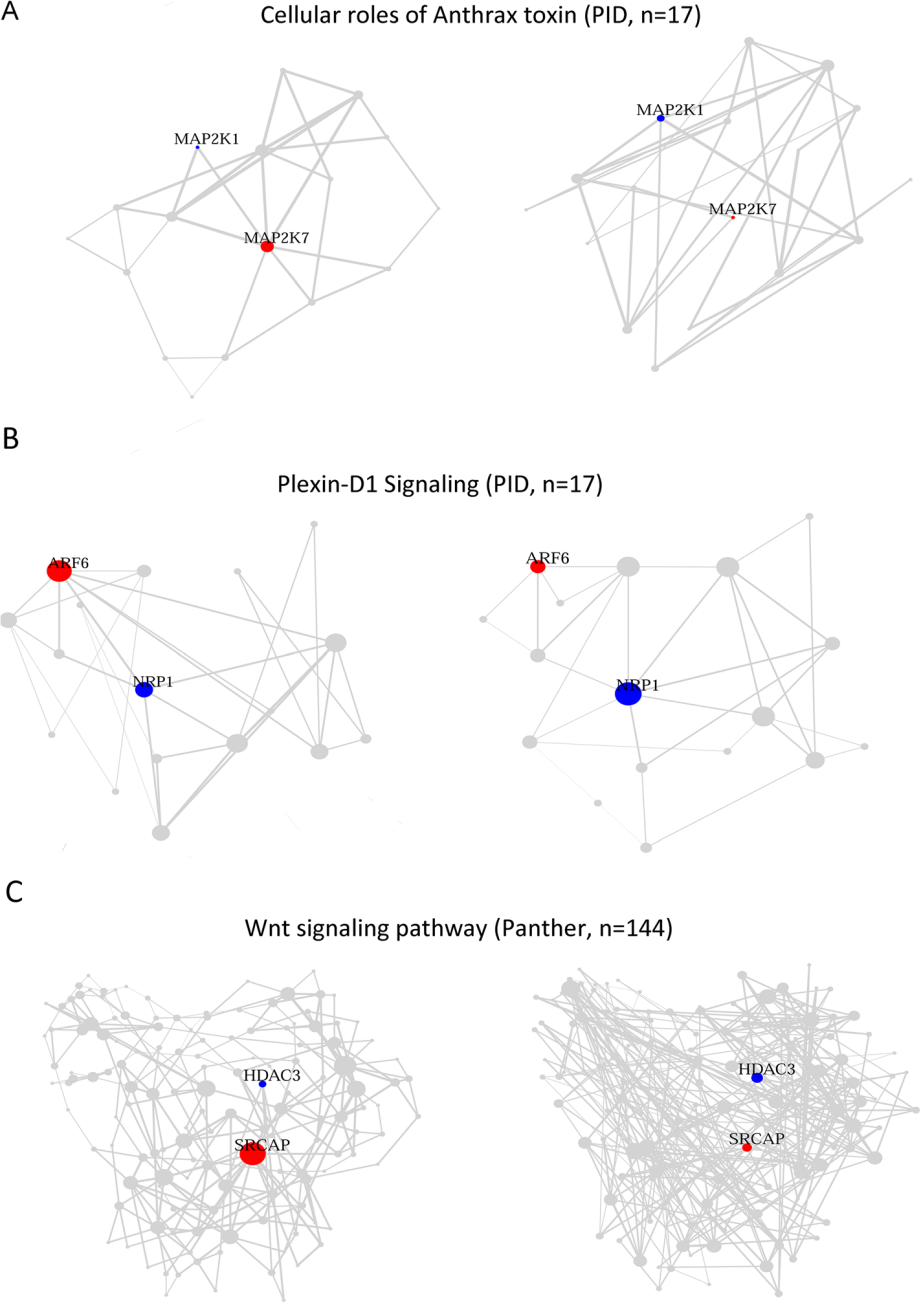
Three genes lost hub status in transcriptome rewiring of their respective pathways in CKD advancement. In each panel, left denotes early CKD and right denotes late CKD. **a** *MAP 2 K7*. **b** *ARF6*. **c** *SRCAP*. Red, hub genes in early CKD. Blue, hub genes in late CKD. Node size, vertex degree. Edge width, absolute correlation

Among those 43 vanishing hubs, 9 were associated with significantly rewired pathways (Table 2), and three of which, *ARF6, MAP 2 K7*, and *SRCAP*, dictated one or multiple focused pathways. *MAP 2 K7* and *ARF6* happen to be the 1st and 2nd most pleiotropic differentially coexpressed genes by virtue of playing the hub role in seven and six pathways (Table 2), respectively, including *Cellular roles of Anthrax toxin* (Fig. 4a) and *Plexin-D1 Signaling* (Fig. 4b). *SRCAP* belonged to only one pathway, *Wnt signaling pathway* (Fig. 4c), serving as its vanishing hub in CKD advancement. Sporadic researches began to imply potential implication of *MAP 2 K7* in hypertensive nephropathy [36, 37] and *ARF6* in diabetic kidney disease [38], respectively.

**Table 2:** Nine differentially co-expressed genes which lost their hub statuses in one or multiple pathways

Gene	DCL rate^**a**^	p	q	Hub incidence	Pathways endorsing hub status
*AKR1C3*	40%	3.52E-40	1.05E-38	1	Prostaglandin and Leukotriene metabolism
*ARF6*	18%	2.58E-17	4.25E-16	6	Alpha4 beta1 integrin signaling events; Arf6 downstream pathway; Arf6 trafficking events; Integrin signaling pathway; Plexin-D1 Signaling; Posttranslational regulation of adherens junction stability and disassembly
*COL6A1*	48%	6.79E-152	1.11E-149	3	Beta1 integrin cell surface interactions; Integrin signaling pathway; Syndecan-1-mediated signaling events
*HCK*	16%	5.70E-08	6.19E-07	1	Glypican 1 network
*ITPR1*	14%	0.00019113	0.0015595	4	Alpha adrenergic receptor signaling pathway; Angiotensin_II-stimulated_signaling_through_G_proteins_and_beta-arrestin; GPCR GroupI metabotropic glutamate receptor signaling pathway; Muscarinic acetylcholine receptor 1 and 3 signaling pathway
*MAP 2 K7*	36%	3.18E-70	1.40E-68	7	Cellular roles of Anthrax toxin; FAS (CD95) signaling pathway; IL-1 signaling pathway (through JNK cascade); IL-1 signaling pathway (through p38 cascade); Toll-like receptor signaling pathway (p38 cascade); Toll-like receptor signaling pathway (through ECSIT, MEKK1, MKKs, JNK cascade); Toll-like receptor signaling pathway (through ECSIT, MEKK1, MKKs, p38 cascade)
*SRCAP*	36%	5.61E-97	3.88E-95	1	Wnt signaling pathway
*TYK2*	27%	9.00E-37	2.47E-35	1	IL-12 signaling (JAK2 TYK2 STAT4)
*VAV2*	16%	3.41E-08	3.83E-07	6	CDC42 signaling events; E-cadherin signaling in the nascent adherens junction; EPHA2 forward signaling; Nectin adhesion pathway; Regulation of CDC42 activity; Regulation of RAC1 activity

^a^ proportion of Differentially Coexpressed Links (DCLs) in total incident links

### Disrupted pathway crosstalk in CKD advancement

**Fig. 5:**
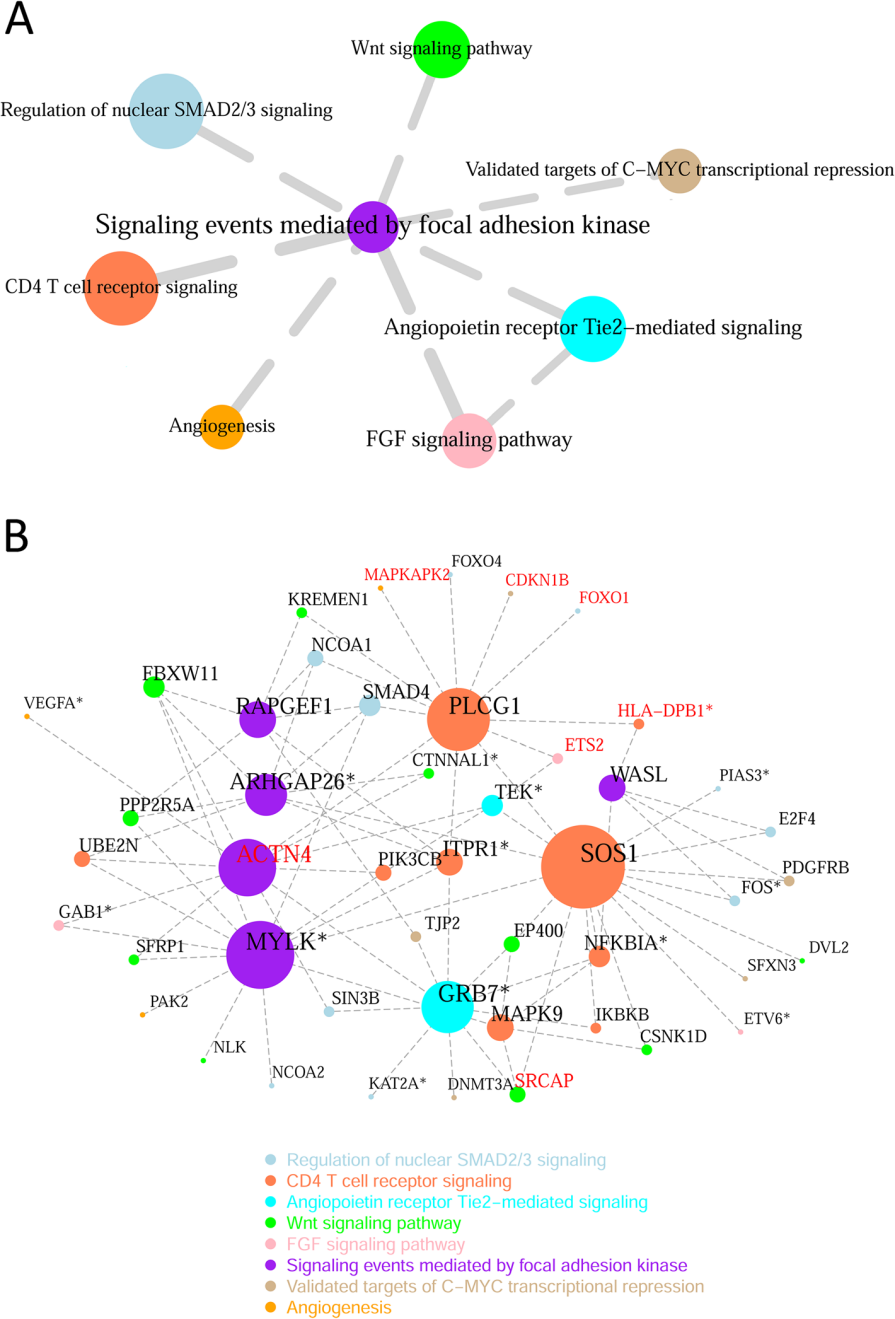
Disruption of pathway crosstalk in CKD progression. **a** pathway crosstalks present in early CKD were disrupted in late CKD. Node size and edge width are proportional to the statistical significance of correlation loss (extremity of *p* value). **b** attenuated cross-pathway gene correlation links incident to the affected pathways. For clarity, only links pertaining to Differentially Coexpressed Genes or hub genes were shown. Node size, vertex degree. Node color, pathway membership. Red text, pathway hub genes. Asterisk (*), differentially expressed genes (FDR < 0.3)

We deployed the 31,384 correlation-decreased differentially coexpressed links from the RNA-seq dataset into a global gene-gene connection network, upon which we sought to identify significantly weakened pathway-pathway connections. At a *p* < 0.05 criterion, eight connections among eight focused pathways were deemed significantly weakened in CKD advancement, which formed a disrupted pathway crosstalk map centered on *Signaling events mediated by focal adhesion kinase* (Fig. 5a). As a verification attempt, we repeated the same procedure in the union network of decreased gene links from all three datasets, which comprised 15,834 more edges (50% in addition) than the RNA-seq-derived network. Ten edges connecting ten pathways emerged in the verification analysis (Additional file 1: Fig. S3), which shared seven nodes and seven edges with the primary finding herewith. In both crosstalk maps, *Signaling events mediated by focal adhesion kinase* was placed at the centric position, indicating that most disrupted crosstalk flows had revolved around it. Focal adhesion kinase (*PTK2*) is a critical regulator of cell movement and is implicated in TGF-beta signal transduction in CKD [39]. In our pathway compendium, *Signaling events mediated by focal adhesion kinase* consists of 53 genes derived from PID, sharing only two members with the 71 genes of *Regulation of nuclear SMAD2/3 signaling*. A total of 73 cross-pathway correlation links accounted for the disrupted crosstalk between *Signaling events mediated by focal adhesion kinase* and *Regulation of nuclear SMAD2/3 signaling* (Additional file 2: Table S3)*.*

The significant disruption of the individual pathway-pathway crosstalks was attributed to correlation-attenuated gene pairs traversing pathway boundaries (Additional file 1: Fig. S4), where ~ 42% edges pertained to *Signaling events mediated by focal adhesion kinase*. For enhanced legibility, we delineated a subnetwork of these cross-pathway correlations involving differentially coexpressed genes or hub genes only (Fig. 5b). In this gene-gene correlation disruption network, 44 of 83 total edges were connected to five differentially coexpressed genes from *Signaling events mediated by focal adhesion kinase*, namely *ACTN4*, *ARHGAP26, MYLK, RAPGEF1*, and *WASL*. As the hub of *Signaling events mediated by focal adhesion kinase* in early CKD, *ACTN4* lost its hub status in late CKD (Table 1), and decreased its correlation with genes from all seven linked pathways but *Validated targets of C-MYC transcriptional repression*. *ACTN4* is genetically associated with focal segmental glomerulosclerosis in OMIM, and it is included in a very recent kidney-disease gene panel towards a comprehensive genetic diagnosis of cystic and glomerular inherited kidney diseases [40]. Eleven direct neighbors, *CTNNAL1, FBXW11, GAB1, GRB7, PIK3CB, PLCG1, SFRP1, SIN3B, TEK, UBE2N*, and *VEGFA*, disassociated themselves with *ACTN4* in CKD advancement. Compared with *ACTN4, SOS1, MYLK*, and *PLCG1* have even greater numbers of connections in the cross-pathway gene-gene correlation disruption network. These three genes haven’t been directly associated with CKD but were implicated in IgA nephropathy (*SOS1*) [41], diabetic kidney disease (*MYLK*) [38], and paroxysmal nocturnal hemoglobinuria, respectively (*PLCG1*) [42].

## Discussion

Compared to the conventional differential expression approach, differential coexpression analysis represents a different yet complementary perspective into diseased transcriptomes. Methods purposed for identification of differentially coexpressed genes, gene connections, and gene sets have been invented and improved in nearly two decades. While a negative voice questioned the potential confounding between differential expression and differential coexpression [43], more studies [18, 44–47] proved that differential coexpression dissection of transcriptomes led to unique, innovative discoveries otherwise invisible to the conventional differential expression approach. In our analysis, differentially coexpressed genes effectively enriched clinically actionable genes (as included in a kidney-disease panel of 140 genes [40]) two times more likely than random embedding. Generally speaking, differential coexpression approaches are enjoying steadily increasing appreciation and are frequently playing a critical role in studies of dysfunctional mechanisms of human disease [1, 10].

In this work, we applied a variety of differential coexpression oriented methods to analyze the transcriptome transition from early stage CKD patients to late stage CKD patients, making innovative discoveries at multiple levels covering vanishing hub genes, disassociated gene links, and disrupted pathway crosstalks. Our results recapitulated well-known CKD pathways such as *Regulation of nuclear SMAD2/3 signaling* and *Signaling events mediated by focal adhesion kinase* and highlighted critical genes such as *ACTN4* in the context of transcriptome correlation network. Plenty of researches have proved the role of Smad2/3 in kidney disease. Smad2 and Smad3, two major downstream mediators of transforming growth factor-β1 (TGF-β1), play a dominant role in kidney dysfunction and renal fibrosis [48]. Smad2 and Smad3 are activated in kidneys of patients with CKD and experimental animals of unilateral ureteral obstruction (UUO), 5/6 nephrectomy, hypertensive nephropathy and diabetic nephropathy [49–54]. Smad2 and Smad3 mediate the transcription of many extracellular matrix (ECM) proteins including connective tissue growth factor, fibronectin and various collagens [55, 56]. The activation of Smad2 and Smad3 results in aggressive ECM deposition in interstitial and glomerulus that cause interstitial fibrosis and glomerulosclerosis in kidney respectively [56], while the inhibition of Smad3 phosphorylation retarded renal fibrosis in UUO rats [57]. Knock-down of Smad3 in mice significantly attenuated renal fibrosis in diabetic nephropathy [58] and aristolochic acid-induced nephropathy [59].

In the present study, we identified *Signaling events mediated by focal adhesion kinase* as the pathway centered on the disassembled pathway crosstalk network during CKD progression. *PTK2*, a non-receptor tyrosine kinase, is one of the first molecules recruited to focal adhesions in response to external mechanical stimuli that controls cell migration, cell proliferation and cell survival in kidney [60, 61]. *PTK2* has the ability to regulate AKT, PI3K, MAPK, Yes-associated protein, integrin α, TGF-β1 and α-smooth muscle actin (α-SMA) [60–63]. ECM accumulation triggers the phosphorylation and recruitment of *PTK2* to focal adhesions [64]. In addition, *PTK2* promoted the expression of monocyte chemoattractant protein-1 and cell migration to accelerate the progression of various glomerular diseases [65]. Interestingly, *PTK2* was required for phosphorylation of *ACTN4,* a vanishing hub gene with substantial contribution to pathway crosstalk disruption in CKD progression*. ACTN4* is closely associated with CKD, especially focal segmental glomerulosclerosis (FSGS) [66, 67]. Mutations in *ACTN4* has been identified as a cause of familial focal segmental glomerulosclerosis [68], and the differences between patients and families who harbor *ACTN4* mutations can be distinguished from other podocyte diseases [69]. Podocytes isolated from mutant *ACTN4* knock-in mice developed extensive and irrecoverable reductions compared with those isolated from wild type mice, and mutant cells were more likely to detach upon stretch [70]. In addition, *ACTN4* can enhance NF-κB activity in podocytes which aggravates podocyte injury [71].

Additional pathways and genes in our results may provide worthwhile research targets in future CKD studies. The disassociated gene links and disrupted pathway crosstalks identified by analyses, such as those gene links revolving around *ACTN4* and those pathway connections incident to *Signaling events mediated by focal adhesion kinase*, may propel specific biological hypotheses on CKD molecular mechanisms.

Notably, we discovered a global expression correlation attenuation within and between key signaling pathways in CKD progression. A similar trend of global loss of transcriptome correlation was previously observed in aging mice [72]. Moreover, a most recent study [73] found genetic and environmental perturbations on human subjects tended to cause universal attenuation of transcriptome coherence. The authors investigated both metabolism and transcriptome data of a variety of perturbation factors, including internal genetic variants and external environmental stresses, and they repeatedly discovered a widespread decrease in the magnitude of pairwise correlation coefficients between mRNA transcripts or metabolites. They referred to this loss of correlation in an infected or diseased state, relative to a baseline or healthy state, as ‘decoherence’. Global correlation loss, or regulatory decoherence, seems to be compatible with the evolutionary perspective of decanalization [74], which hypothesizes that new mutations or novel environments may almost inevitably disrupt the fine-tuned gene regulatory network resulting from many generations of stabilizing selection. Taking into account both the present work and previous related studies, the pattern of global correlation losses have been noted in aging, immune challenge, metabolic disease, and CKD. It is intriguing to investigate if such a global correlation loss trend exists in extended pathological scenarios.

## Conclusions

In this study, we performed RNA-Seq transcriptome profiling of five stages of chronic kidney disease patients and analyzed the transcriptome correlation disruptions accompanying CKD progression in the context of signaling pathways using a combination of differential coexpression methods. Overall, a global expression correlation attenuation was observed in CKD progression, with pathway *Regulation of nuclear SMAD2/3 signaling* demonstrating the most remarkable intra-pathway correlation rewiring. We identified 27 focused pathways that significantly changed the internal gene-gene expression correlation in CKD advancement, and enumerated 44 presumably CKD-relevant genes on account of their vanishing hub roles in the collapsed pathways. Moreover, we went further to delineate a disrupted pathway crosstalk map centered upon *Signaling events mediated by focal adhesion kinase*; well-known relevant genes (such as *ACTN4*) and relevant pathways (such as *Regulation of nuclear SMAD2/3 signaling*) were found involved in these inter-pathway disassociations.

## Supplementary information

**Additional file 1: Figure S1.** Universal correlation attenuation within focused pathways. All focused pathways listed in Table 1, except eight depicted in Fig. 2, are illustrated here. Rows and columns represent genes of the concerned pathway, arranged in identical order. Cells denote the expression correlation values between the row gene and the column gene, with the lower triangle and the upper triangle indicating the early CKD and late CKD phenotypes, respectively. **Figure S2.** Fourteen vanishing hub genes had statistically significant differential expression between CKD stages (FDR< 0.3). Differential expression analysis was performed via Comulative Link Models for ordinal regression. **Figure S3.** Disrupted pathway crosstalk map inferred from the union network of decreased gene links from all three datasets. The background gene-gene network comprised 47,218 correlation-loss edges. Node size and edge width are proportional to the statistical significance of correlation loss (extremity of p value). Each edge was labelled with the p value out of CSPN analysis. **Figure S4.** Correlation-attenuated gene pairs traverse pathway boundaries shedding light on disrupted pathway crosstalks. Figure 4b forms a sub-graph of the present network.

**Table S1.** Clinical and demographic baseline characteristics of controls and patients with CKD. Results are expressed as the means ± SD, *P< 0.05, **P< 0.01. **Table S2.** Except GCLM, all 44 hub genes in early CKD pathway-wise co-expression networks lost their hub status in late CKD. These genes were identified as differentially coexpressed genes by DCGL as well. **Table S3.** Correlation-attenuated gene pairs that traverse pathway boundaries.

## Data Availability

The data that support the findings of this study are available from the corresponding author upon reasonable request. Majority of the data analyses were performed using R × 64 3.4.2. All R codes written for this manuscript are available from the corresponding author upon request.

## Abbreviations

CKD: chronic kidney disease
CSPN: Characteristic Sub-Pathway Network
DCGL: Differentially Coexpressed Genes and Links
eGFR: estimated Glomerular Filtration Rate
FDR: False Discovery Rate
GSNCA: Gene Sets Net Correlations Analysis
WGCNA: Weighted Gene Correlation Network Analysis
