# Supplementary material for: Expression correlation attenuates within and between key signaling pathways in chronic kidney disease
Source: BMC Med Genomics. 2020 Sep 21;13(Suppl 9):134. doi: 10.1186/s12920-020-00772-3 (PMC7504859; doi:10.1186/s12920-020-00772-3)

**Expression correlation attenuates within and between key signaling pathways in chronic kidney disease**

Hui Yu^1^, Danqian Chen^2^, Olufunmilola Oyebamiji^1^, Ying-Yong Zhao^2*^, Yan Guo^1*^

In this **Additional file 1**, we present four supplementary figures: Figures S1-S4.

**Figure S1.** **Universal correlation attenuation within focused pathways.** All focused pathways listed in Table 1, except eight depicted in Figure 2, are illustrated here. Rows and columns represent genes of the concerned pathway, arranged in identical order. Cells denote the expression correlation values between the row gene and the column gene, with the lower triangle and the upper triangle indicating the early CKD and late CKD phenotypes, respectively.

**Figure S2. Fourteen vanishing hub genes had statistically significant differential expression between CKD stages (FDR<0.3).**

**Figure S3. Disrupted pathway crosstalk map inferred from the union network of decreased gene links from all three datasets.** The background gene-gene network comprised 47,218 correlation-loss edges. Node size and edge width are proportional to the statistical significance of correlation loss (extremity of p value). Each edge was labelled with the p value out of CSPN analysis.

**Figure S4. Correlation-attenuated gene pairs traverse pathway boundaries shedding light on disrupted pathway crosstalks.** Figure 4B forms a sub-graph of the present network.

**Figure S1**


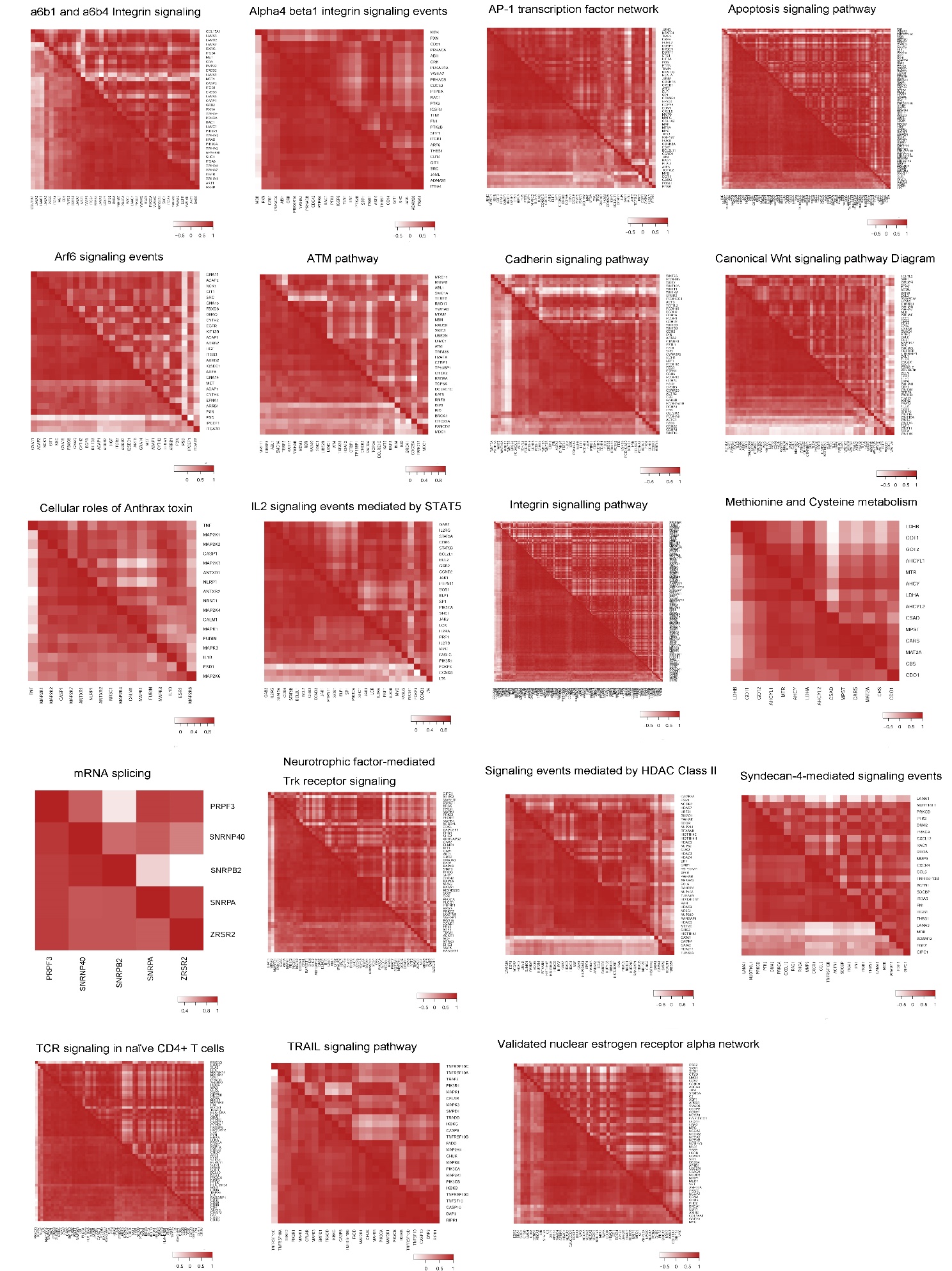


**Figure S2**


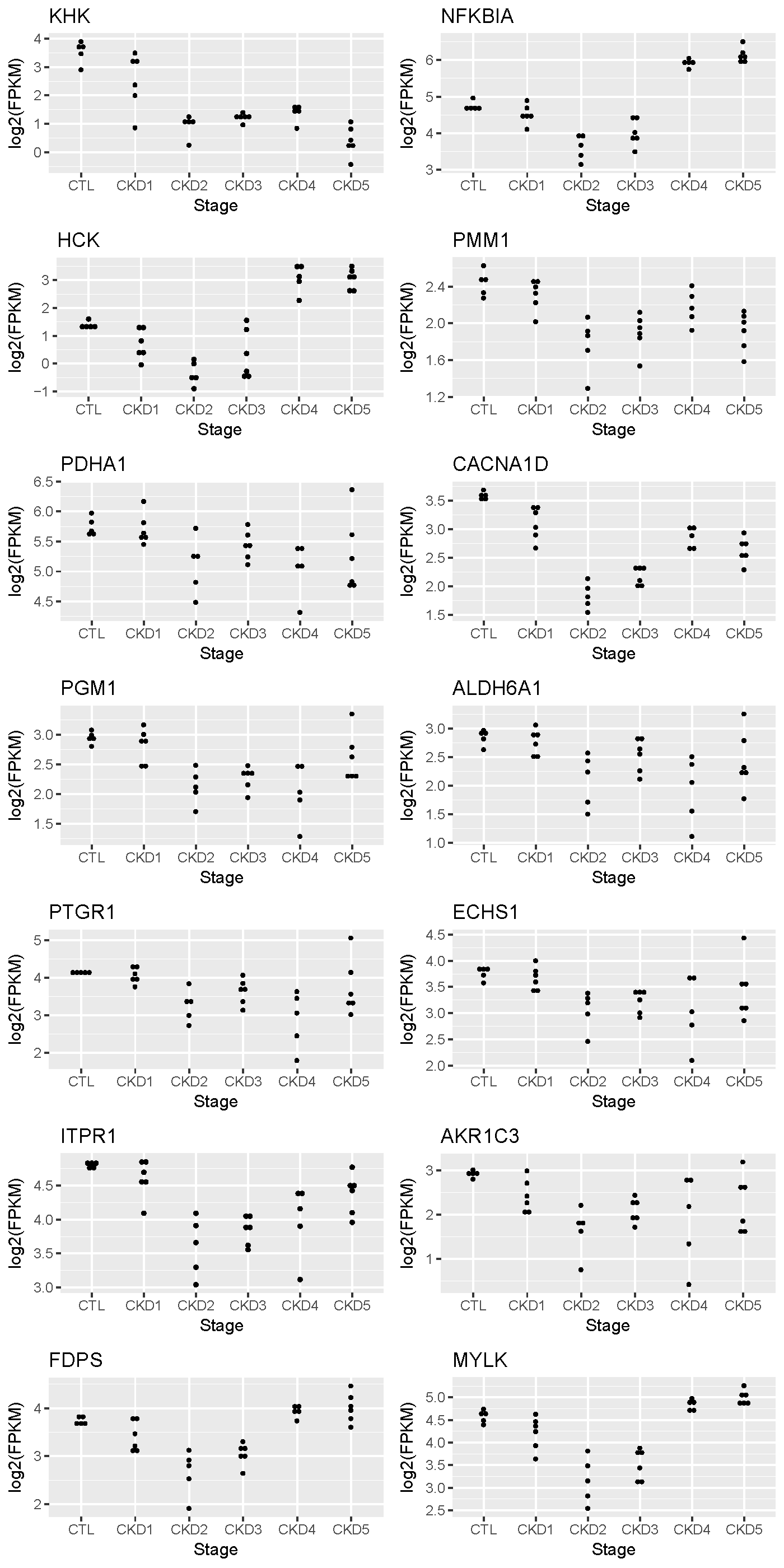


**Figure S3**


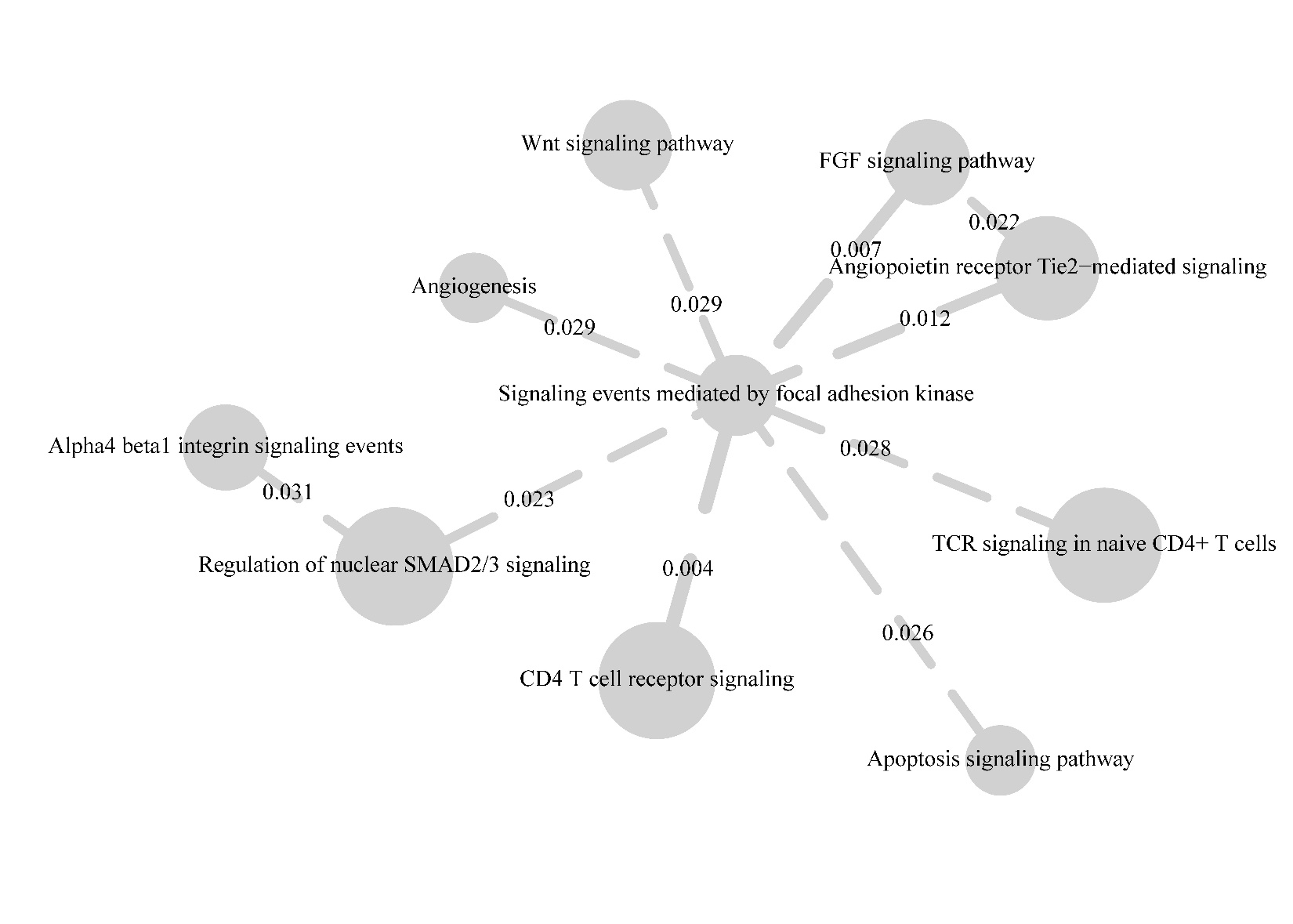


**Figure S4**


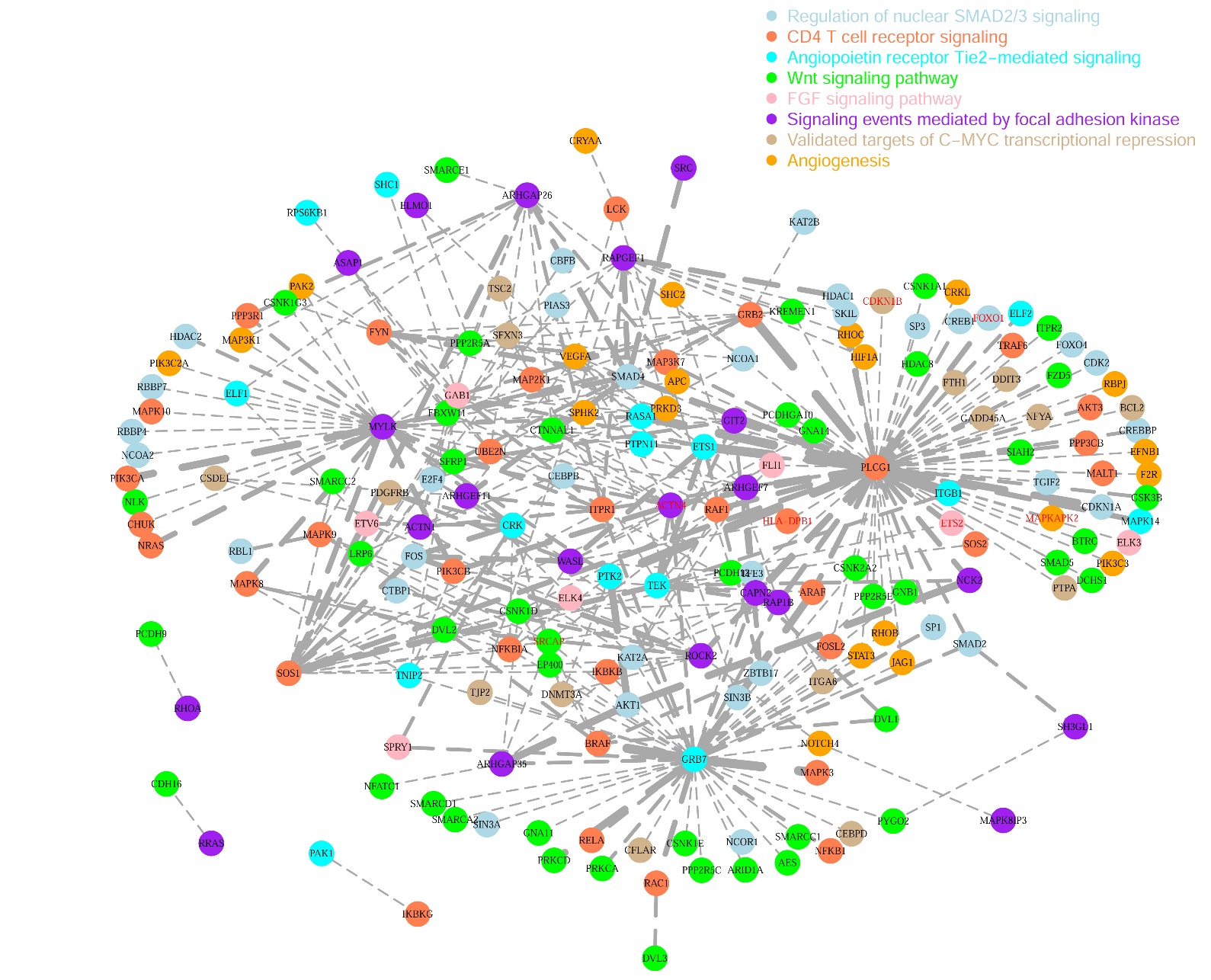

Supplement: Supplementary file 1 — Additional file 1: Figure S1. Universal correlation attenuation within focused pathways. All focused pathways listed in Table 1, except eight depicted in Fig. 2, are illustrated here. Rows and columns represent genes of the concerned pathway, arranged in identical order. Cells denote the expression correlation values between the row gene and the column gene, with the lower triangle and the upper triangle indicating the early CKD and late CKD phenotypes, respectively. Figure S2. Fourteen vanishing hub genes had statistically significant differential expression between CKD stages (FDR< 0.3). Differential expression analysis was performed via Comulative Link Models for ordinal regression. Figure S3. Disrupted pathway crosstalk map inferred from the union network of decreased gene links from all three datasets. The background gene-gene network comprised 47,218 correlation-loss edges. Node size and edge width are proportional to the statistical significance of correlation loss (extremity of p value). Each edge was labelled with the p value out of CSPN analysis. Figure S4. Correlation-attenuated gene pairs traverse pathway boundaries shedding light on disrupted pathway crosstalks. Figure 4b forms a sub-graph of the present network. [file 12920_2020_772_MOESM1_ESM.docx]
